# Intratumoral Immunotherapy with Cowpea Mosaic Virus and Interleukin-12 Eliminates Established Treated Tumors and Generates Robust Systemic Immunity to Suppress the Growth of Untreated Metastatic Tumors

**DOI:** 10.1021/acs.molpharmaceut.4c01539

**Published:** 2025-06-06

**Authors:** Alex Misiaszek, Alicia M. Santos, Greg W. Ho, Jennifer Fields, Kevine Silihe Kamga, Radu Stan, Jessica F. Affonso De Oliveira, Nicole F. Steinmetz, Steven Fiering

**Affiliations:** † Department of Microbiology and Immunology, 12285 Dartmouth Geisel School of Medicine, Hanover, New Hampshire 03755, United States; ‡ Dartmouth Cancer Center, Dartmouth Geisel School of Medicine, Hanover, New Hampshire 03755, United States; Aiiso Yufeng Li Family Department of Chemical and Nano Engineering, Center for Nano-ImmunoEngineering, Shu and K.C. Chien and Peter Farrell Collaboratory, Center for Engineering in Cancer, Moores Cancer Center, Department of Bioengineering, Department of Radiology, University of California San Diego, 9500 Gilman Dr., La Jolla, California 92093, United States; ▼ Department of Biochemistry and Cell Biology, Dartmouth Geisel School of Medicine, Hanover, New Hampshire 03755, United States

**Keywords:** cowpea mosaic virus, IL-12, intratumoral immunotherapy, *in vivo* transfection, electroporation

## Abstract

Intratumoral immunotherapy (ITIT) delivers immune-stimulating agents or treatments directly into solid tumors to reverse the immunosuppressive nature of the tumor microenvironment (TME) and enables local and systemic antitumor immunity. However, optimal clinical strategies for ITIT are not yet fully understood. Cowpea mosaic virus (CPMV) is a plant virus that is not infectious in mammals but is a powerful multi-toll-like receptor (TLR) agonist which stimulates potent antitumor immunity when used in ITIT. ITIT by transfection of interleukin-12 (IL-12) expressing plasmids into melanoma tumors *via* *in vivo* electroporation is being assessed in Phase II clinical trials to treat metastatic disease. We investigated the combination of CPMV with IL-12 plasmid electroporation to generate systemic (abscopal) immunity superior to that from either monotherapy alone. Mice were inoculated with two B16–F10 melanoma or MC38 colorectal cancer tumors and one tumor on each flank. One tumor was treated once per week for 2 weeks with IL-12 electroporation and CPMV intratumoral injection. IL-12 electroporation following CPMV injection greatly reduced the efficacy of CPMV by disrupting its viral structure. Conversely, electroporation of IL-12 prior to CPMV injection led to total clearance of 100% of treated tumors and significantly greater suppression of distant, untreated tumors compared to either monotherapy or the reverse treatment order (CPMV IT injection, followed by IL-12 electroporation). This superior abscopal effect led to a significantly improved survival. Our novel treatment strategy offers significant translational value in treating solid tumors by improving the local and systemic efficacy of IL-12 electroporation or CPMV alone.

## Graphical abstract

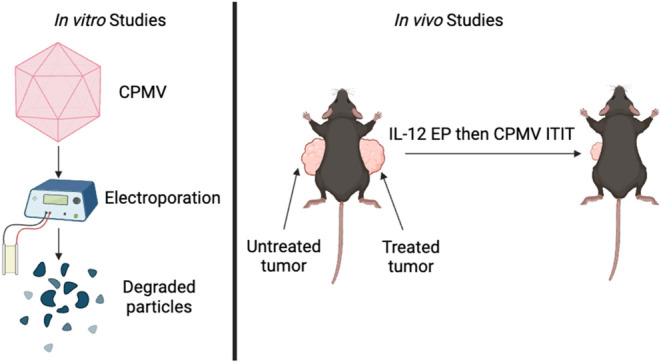

## Introduction

Cancer immunotherapies stimulate the immune system to recognize and eliminate malignant cells.^1^ To be effective, these therapies must overcome the immunosuppressive nature of the tumor microenvironment (TME) which protects tumors from antitumor immunity.^2^ Intratumoral immunotherapy (ITIT) is a promising method of generating antitumor immunity that is currently being actively investigated and clinically utilized. ITIT involves administration of immunostimulatory agents or treatments directly into solid tumors which reverses the local tumor-mediated immune suppression of the TME, converting it into a proinflammatory environment.^3^ Immune stimulation leads to recruitment of various immune effector cells, including cytotoxic T lymphocytes (CTLs) into the tumor which become primed against the whole suite of tumor antigens, mediating antitumor immunity against both the treated tumors and distant metastatic tumors.^4^

We have previously demonstrated the effectiveness of the plant pathogen, cowpea mosaic virus (CPMV), as a powerful immune stimulator (immune adjuvant) for ITIT that generates antitumor protection in murine mouse models and canine cancer patients (pets) for both treated local and untreated distant tumors (the “abscopal effect”).^5,6^ CPMV is a powerful toll-like receptor (TLR) 2, 4, and 7 agonist which causes a significant inflammatory response.^7^ Notably, CPMV is distinct from oncolytic viruses that infect cells, replicate, and lyse cells, because it does not replicate in mammalian cells.^8^ TLR activation by CPMV signals through myeloid differentiation primary response 88 (MYD88) to activate immune cells and drives expression of proinflammatory cytokines including type-I interferons and interferon-γ (IFN-γ).^5^ While the local antitumor effect of CPMV works primarily through innate immune cells, it also generates improved CTL responses to drive systemic antitumor immunity. However, CPMV does not consistently generate IL-12, a primary activator of CTLs,^9^ in previous *in vitro* studies^5^ or unpublished *in vivo* studies. We therefore hypothesized that coadministering CPMV with IL-12 would amplify CTL-mediated immunity and improve both local and systemic antitumor efficacy.

IL-12 has been investigated as a potential immunotherapeutic treatment.^10^ IL-12 drives a Th1-skewed immune response which recruits CTLs to eliminate cancer cells.^9^ Early clinical trials administering IL-12 intravenously were limited in their success since systemic IL-12 administration caused serious adverse events.^9,11–13^ Despite these toxicities, a phase II clinical trial reported potential benefits of intravenous administration of IL-12.^14^ This study reported that 40% of patients with Hodgkin’s lymphoma had a partial or full response to intravenous IL-12 administration. Further investigation into the therapeutic use of IL-12 revealed that toxicity was dependent on high systemic levels of IL-12 and that intratumoral administration with high local but low systemic levels was tolerable.^15^ In particular, it was demonstrated in a phase IB clinical trial that intratumoral injection of IL-12 expressing plasmid led to a reduction in treated tumor size in 30% of patients without elevated serum levels of proinflammatory cytokines or associated adverse events.^15^

*In vivo* plasmid electroporation for ITIT has been investigated to deliver immune stimulatory proteins to tumors.^16^ This approach involves intratumoral injection of plasmid DNA followed by pulses of electricity delivered by electroporation that create transient membrane holes and mediates transfer of plasmids into cells, where transcription and protein expression can occur.^17,18^ Plasmid electroporation of IL-12 was not only safe, with minimal adverse events, but also had a strong signal of efficacy in a phase I clinical trial of 19 patients with metastatic melanoma where 10% of patients experienced complete clearance of metastatic untreated lesions and an additional 42% experienced partial clearance or disease stabilization.^19^ IL-12 plasmid electroporation had further success in a phase II clinical trial for metastatic melanoma where 17.9% of patients experienced complete regression of treated tumors, 46% had regression in at least one untreated lesion, and 25% demonstrated net regression across all untreated lesions.^20^ The FDA has approved an *in vivo* electroporation device that could be used to electroporate plasmids, supporting the potential of *in vivo* electroporation as a cancer therapy for humans.^21^

ITIT by either CPMV or IL-12 plasmid electroporation inhibits the growth of treated tumors and generates increased immune pressure that slows the growth of untreated tumors in mice. Although CPMV’s abscopal effect is mediated by T-cell immunity, it does not consistently induce IL-12 production; thus, combining it with IL-12 plasmid electroporation may enhance T-cell activation and improve the abscopal response. Given the efficacy of these single-agent ITIT, we combined the two treatments with the goal of generating increased local and abscopal antitumor immunity.

## Materials and Methods

### Materials

CPMV was propagated and purified from infected cowpeas as previously described; purified CPMV was stored frozen in 0.1 M potassium phosphate (KP) pH 7.0 buffer as previously described.^22^ IL-12 plasmid expresses both IL-12 P35 and P40 driven by the cytomegalovirus (CMV) enhancer/promoter.^23^ The plasmid was a gift from Shulin Li of the MD Anderson Cancer Center.

### Plasmid Purification

IL-12 plasmid was amplified in overnight cultures and purified using the QIAGEN Plasmid *Plus* Maxi Kit according to the manufacturer’s protocol and resuspended in endonuclease-free water. Plasmid concentration was determined by a NanoDrop spectrophotometer.

### Spleen Processing

Spleens were harvested from 6–8-week-old female C57B/6 mice and immediately placed into warm RPMI media. Spleens were subsequently mashed through a 70 μm cell strainer, centrifuged at 350*g* for 5 min at 4 °C, and treated with RBC lysis buffer from Biolegend at room temperature for 10 min. The lysis reaction was then stopped by the addition of excess volume of PBS. Cells were centrifuged at 350*g* for 5 min at 4 °C and resuspended in RPMI media supplemented with 10% FBS, 2 mM l-glutamate, and 0.05% penicillin/streptomycin (complete media). Cells were stained and counted with trypan blue to assess their viability.

### Splenocyte Stimulation

500,000 splenocytes were aliquoted in 200 μL in ThermoFisher 96-well round-bottom Nunc plates in complete RPMI. Experimental samples were stimulated with 2.5 μg of native or electroporated CPMV suspended in 2.5 μL of complete 0.01 mM potassium phosphate (KP) buffer. 2.5 μL of complete RPMI containing no CPMV was added to negative control wells. Stimulation occurred for 24 h at 37 °C with 5% CO_2_. Cultures were centrifuged at 350xg for 5 min at 4 °C after 24 h, and supernatants were removed. Supernatants were stored at −20 °C.

### ELISA

ELISAs were carried out using Biolegend IFN-α1, IFN-γ, IL-6, and IL-12 kits according to the manufacturer’s protocol in 96-well Nunc MaxiSorp uncoated ELISA plates. Splenocyte supernatants were diluted 1:3 using an assay diluent buffer. Color was developed using a biotinylated streptavidin reporter enzyme with the substrate TMB. Signal intensity was measured as a difference between absorbance at 450 and 570 nm, and concentration was determined based on sample standards.

### *In Vitro* CPMV Electroporation

CPMV was electroporated *in vitro* using a Gene Pulser Xcell Electroporator with 1 ms pulses of 1500 V consistent with *in vivo* electroporation parameters. CPMV was diluted to a concentration of 1 mg/mL in 0.01 M KP buffer for electroporation.

### Gel Electrophoresis

Gel electrophoresis was carried out in a 1.2% agarose gel made with 0.01 mM KP buffer (and also run in KP). The gel was prestained with ethidium bromide diluted at a 1:10,000 concentration. Samples were prepared using 4 μL of 6× gel loading dye purple (New England Biolabs), 8 μL of 0.1 mM KP, and 12 μL of CPMV at 1 mg/mL. 2 μL of RNase A at 20 mg/mL was added to each sample treated with RNase, and 2 μL of sterile water was added to each sample not treated with RNase A. 15 μL of each sample was loaded into the gel. Electrophoresis was carried out in chilled 0.01 mM KP as a running buffer for 40 min at 80 V, 300 mA. The gel was imaged under UV (EtBr setting) for nucleic acid. The gel was then soaked in 25 mL of shrinking buffer (50% MeOH, 42.5% water, 7.5% acetic acid v/v) for 5 min. Shrinking buffer was removed, and 25 mL of destaining buffer (87.5% water, 5% MeOH, 7.5% acetic acid (all v/v)) was added, followed by 2.5 mL of Coomassie stock solution (0.25% Coomassie (w/v), 10% acetic acid, 45% water, 45% MeOH (v/v)). Gel was allowed to stain overnight with shaking. The gel was imaged with Coomassie settings to visualize the protein stain.

### Transmission Electron Microscopy

10 μL CPMV samples (wildtype and electroporated) diluted to 0.1 mg/mL in sterile water were loaded onto carbon-coated 400 mesh copper grids (Electron Microscopy Science) for 3 min. The sample was wiped off, and the grid was washed two times with sterile water. 2% Uranyl acetate (UA) was added to the edge of the grid for 90 s. Excess UA was removed. TEM images were obtained using a Thermo Helios 5 CX electron microscope at 20,00 kV.

### Cell Lines

B16–F10 cells were cultured in RPMI supplemented with 10% fetal bovine serum (FBS), 2 mM glutamate, 1 mM Na-pyruvate, and 0.05% penicillin-streptomycin. MC38 cells were cultured in DMEM supplemented with 10% fetal bovine serum (FBS), 2 mM glutamate, and 0.05% penicillin-streptomycin. Cells were cultured at 37 °C with 5% CO_2_, removed from plates with trypsin, and evaluated with trypan blue stain to ensure viability.

### Mice

Female C57BL/6 mice were purchased from Charles River Laboratories. Mice were all 6–8 weeks old at the beginning of each experiment. All animals were housed in the Laboratory Animal Resource Vivarium under specific pathogen-free conditions, and all studies were conducted with Institutional Animal Care and Use Committee approval.

### Tumor Inoculation

2 × 10^5^ B16–F10 or MC38 cells were injected intradermally into one or both flanks of 6–8-week-old female C57BL/6 mice. For the two-tumor model, the right flank tumor was injected on day −7 and the left flank tumor was injected on day −4. For the one-tumor model, the right flank was injected on day −10. All injections took place under anesthesia with isoflurane. Treatment began on day 0 once average tumor volume across all mice was 50 mm^3^. Average treated tumor volume was normalized across groups to ensure comparability. Mice were evaluated daily, and tumors were measured every 2 days. End point was determined by tumor volume ((*L* ^2^) x *W*/2) equal to or exceeding 1500 mm^3^.

### *In Vivo* Electroporation

Mice were electroporated with 50 μg of IL-12 plasmid in sterile water or 1× PBS at 1500 v/cm for 6 pulses of 1 ms each with a two-prong needle attachment with 10 mm separation. Electroporation was conducted using a BTX Harvard Gemini X2 Electroporator.

### Treatment of the One-Tumor Model

Treatment was administered once on day 0. All mice were treated with two 15 μL injections for a total injection volume of 30 μL to maintain consistency in the treatment strategy between mice in this study and mice in the two-tumor studies. Mice in the PBS group received 2 × 15 μL intratumoral (IT) injections of 1× PBS. Mice in the PBS + EP group received 2 × 15 μL intratumoral (IT) injections of 1× PBS followed by electroporation. Mice in the CPMV group received 1 × 15 μL injection of 100 μg of CPMV in 1× PBS and 1 × 15 μL injection of PBS. Mice in the CPMV + PBS-EP group received 1 × 15 μL injection of 100 μg of CPMV in 1× PBS followed by 1 × 15 μL injection of 1× PBS followed by electroporation. Mice in the EP + CPMV group received 1 × 15 μL injection of 1× PBS followed by electroporation and then received 1 × 15 μL injection of 100 μg of CPMV in 1× PBS. All mouse experiments were conducted with the approval of the Dartmouth College Institutional Animal Care and Use Committee under protocol 2137 and followed the ARRIVE guidelines.

### Treatment of the Two-Tumor Model

Treatment was administered on days 0 and 7 to the right flank (primary) tumor. All mice were treated with two 15 μL injections for a total injection volume of 30 μL to maintain consistency in all groups. Mice in the PBS group received 2 IT injections of 1× PBS. Mice in the CPMV group received an IT injection of 1× PBS followed by IT injection of 100 μg of CPMV suspended in 1× PBS. Mice in the IL-12 group received an IT injection of PBS followed by IT injection and electroporation of 50 μg of IL-12 plasmid suspended in sterile water. Mice CPMV + IL-12 and IL-12 + CPMV groups received IT injection of 100 μg of CPMV in 1× PBS either prior to or following IT injection and electroporation of 50 μg of IL-12 plasmid suspended in sterile water. All mouse experiments were conducted with the approval of the Dartmouth College Institutional Animal Care and Use Committee under protocol 2137 and followed the ARRIVE guidelines.

### Statistical Analysis

GraphPad V.10.2.2 was used to evaluate statistical tests in this study. Student’s *t* test was used to compare differences between the 2 groups. Tumor growth curves were compared using ANOVA, and survival curves were analyzed using a log-rank (Mantel-Cox) test. Statistical significance is reported with *p* > 0.05 as ns, *p* < 0.05 as *, *p* < 0.01 as **, *p* < 0.001 as ***, and *p* < 0.0001 as ****. All experiments were repeated at least once and representative data for each are reported.

## Results

### Electroporation Must Precede CPMV Treatment to Maintain CPMV Efficacy

We first sought to determine whether CPMV would maintain efficacy upon injection into tumors immediately before or following *in vivo* electroporation. C57BL/6 mice bearing B16–F10 tumors were treated one time with various combinations of CPMV (100 μg) and electroporation of PBS (six 1 ms pulses at 1500 V). Mice were either treated with intratumoral (IT) injection of PBS, electroporation of PBS (PBS + EP), IT injection of CPMV, IT injection of CPMV followed by electroporation of PBS (CPMV + EP), and electroporation of PBS followed by IT injection of CPMV (EP + CPMV). These treatments were applied once to established tumors (50 mm^3^) (Figure 1).

**Figure 1:**
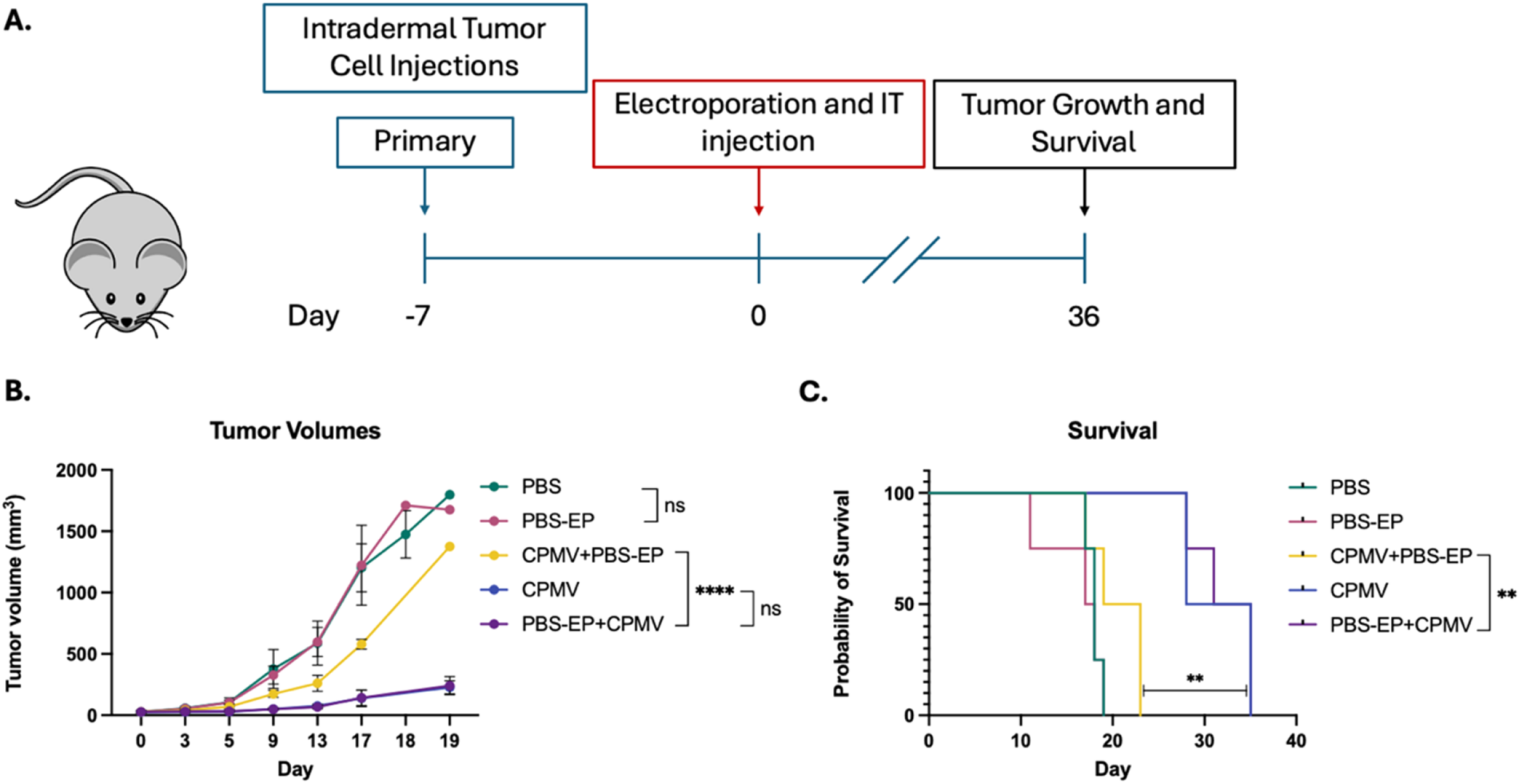
CPMV loses efficacy when exposed to electroporation *in vivo*. (A) Experimental timeline showing the workflow of study. (B) B16–F10 bearing mice treated with electroporation of PBS followed by injection of CPMV (PBS-EP + CPMV) had tumor growth slowed to the same rate as mice treated with CPMV alone, whereas mice treated with CPMV prior to electroporation of PBS (CPMV + PBS-EP) had significantly faster tumor growth. (C) Mice treated with electroporation of PBS followed by injection of CPMV, and mice treated with CPMV alone had comparable probability of survival, which was significantly better than mice treated with CPMV prior to electroporation of PBS. (*N* = 4) Statistical significance is reported with *p* > 0.05 as ns, *p* < 0.05 as *, *p* < 0.01 as **, *p* < 0.001 as ***, and *p* < 0.0001 as ****. This experiment was repeated with *N* = 3, and similar results were observed, validating the results in the full-scale experiment shown here.

Figure 1A shows the treatment timeline for mice in the one-tumor studies. The studies show there is no significant difference in tumor growth (Figure 1B) or probability of survival (Figure 1C) in mice receiving IT PBS (PBS) compared with electroporation of PBS (PBS-EP). This demonstrates that ITIT electroporation with the parameters used for plasmid transfection (six 1 ms pulses at 1500 V) does not induce significant tumor cell death or sufficient inflammation to drive antitumor immunity on its own. CPMV was administered in three different ways: IT injection of CPMV alone (CPMV), IT injection of CPMV followed by electroporation of PBS (CPMV + PBS-EP), or electroporation of PBS followed by IT injection of CPMV (PBS-EP + CPMV). Although electroporation nearly abolished the immune stimulatory ability of injected CPMV when mice are treated with CPMV first followed by electroporation of PBS (CPMV + PBS-EP), CPMV efficacy in slowing tumor growth and improving probability of survival is maintained with no difference comparing CPMV alone *vs* PBS-EP + CPMV. Based on these findings, we hypothesized that CPMV’s loss of antitumor efficacy is because of the effects of electroporation on the virus itself rather than a change in the immune landscape of the TME.

### CPMV Loses Structural Integrity When Exposed to Electroporation *In Vitro*

We evaluated the effect of *in vitro* electroporation on CPMV’s immunogenicity with a splenocyte stimulation assay.^5^ A sample of CPMV was electroporated *in vitro* at a concentration of 1 mg/mL in 0.01 M KP buffer using six 1 ms pulses of 1500 V, the same conditions used to electroporate tumors *in vivo.* Splenocytes were isolated from freshly removed C57BL/6 mouse spleens and then exposed to native CPMV, electroporated CPMV, or buffer only. The final concentration of CPMV in the native and electroporated CPMV stimulation samples was 12 μg/mL, resulting in 500,000 virion particles per splenocyte. After 24 h, supernatants from each cell culture were collected and assayed by enzyme-linked immunosorbent assays (ELISAs) for detection of IFN-α1, IFN-γ, IL-6, and IL-12 (Figure 2).

**Figure 2:**
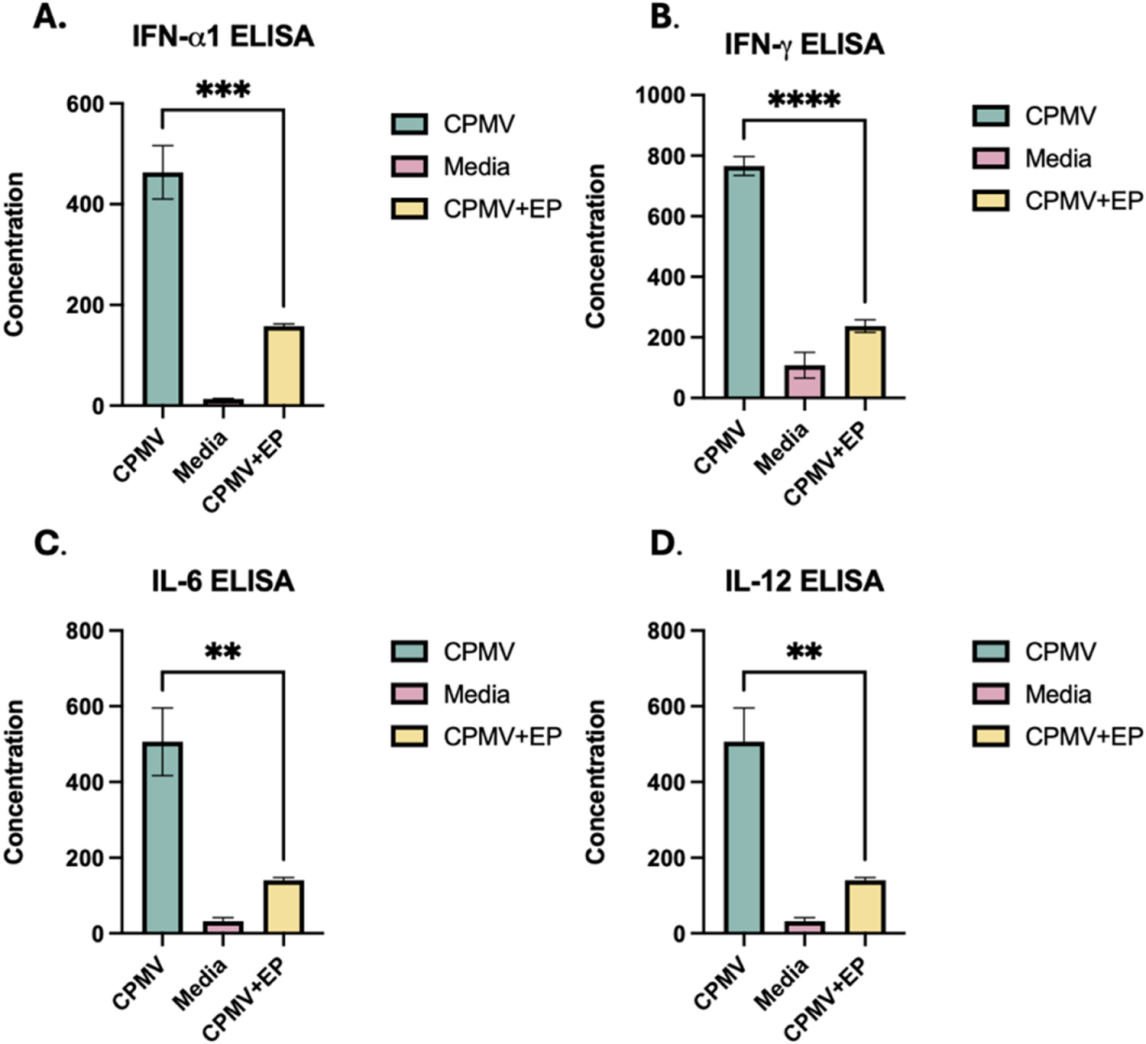
*In vitro* electroporation reduces the immunogenicity of CPMV. ELISAs to detect (A) IFN-α1, (B) IFN-γ, (C) IL-6, and (D) IL-12 in supernatants removed from isolated splenocytes cultured *in vitro* with CPMV, electroporated CPMV, or media show that CPMV’s immunogenicity is significantly diminished after electroporation. Electroporated CPMV maintains a minimal amount of immunogenicity compared to media. Statistical significance is reported with *p* > 0.05 as ns, *p* < 0.05 as *, *p* < 0.01 as **, *p* < 0.001 as ***, and *p* < 0.0001 as ****. Three technical replicates for each condition were individually assayed. The error bars represent the deviation between those replicates. The entire experiment was repeated and generated similar data, which is not shown.

Electroporated CPMV elicited significantly weaker splenocyte activation, as measured by all four cytokines assayed when compared to CPMV without electroporation. Notably, IFN-α1 production, which is activated through TLR-7 signaling, was diminished (Figure 2A). TLR-7 detects ssRNA, so the decrease in the level of production of this cytokine suggests that the RNA contained within the viral capsid is disrupted by electroporation. Moreover, the decrease in production of IFN-γ, IL-6, and IL-12 (Figure 2B,C) suggests that recognition of the protein coat of CPMV by TLRs 2 and 4 is also disrupted.

These findings were consistent with those presented in Figure 1, as CPMV’s immunogenicity is significantly reduced but not completely abolished when exposed to electroporation *in vitro*. Additionally, CPMV’s immunogenicity was diminished in terms of signaling pathways activated by both its protein coat and RNA, suggesting that electroporation diminishes CPMV’s immunogenicity by disrupting its structure. Therefore, we characterized the structural changes in the CPMV caused by electroporation.

### CPMV Is Structurally Unstable When Exposed to Electroporation *In Vitro*

To evaluate the structural integrity of CPMV as a result of electroporation, a titration was conducted increasing the number of pulses of electroporation to which CPMV was exposed *in vitro*. Native CPMV and samples of CPMV electroporated with two, four, six, or eight 1 ms pulses at 1500 V were cultured with isolated splenocytes for 24 h as in Figure 2. The immunogenicity of each sample was evaluated with an ELISA of each culture’s supernatant to detect IFN-γ. This experiment revealed that CPMV maintains its immunogenicity after 2 pulses of electroporation and that its immunogenicity is significantly reduced after 4 pulses, with modest further reduction after 6 or 8 pulses (Figure 3A). Based on this result, we compared native CPMV (CPMV), CPMV electroporated with 2 pulses (2xEP CPMV), and CPMV electroporated with 6 pulses (6xEP CPMV) in subsequent characterization experiments. Virus particle integrity was then analyzed by native gel electrophoresis and transmission electron microscopy (TEM).

**Figure 3:**
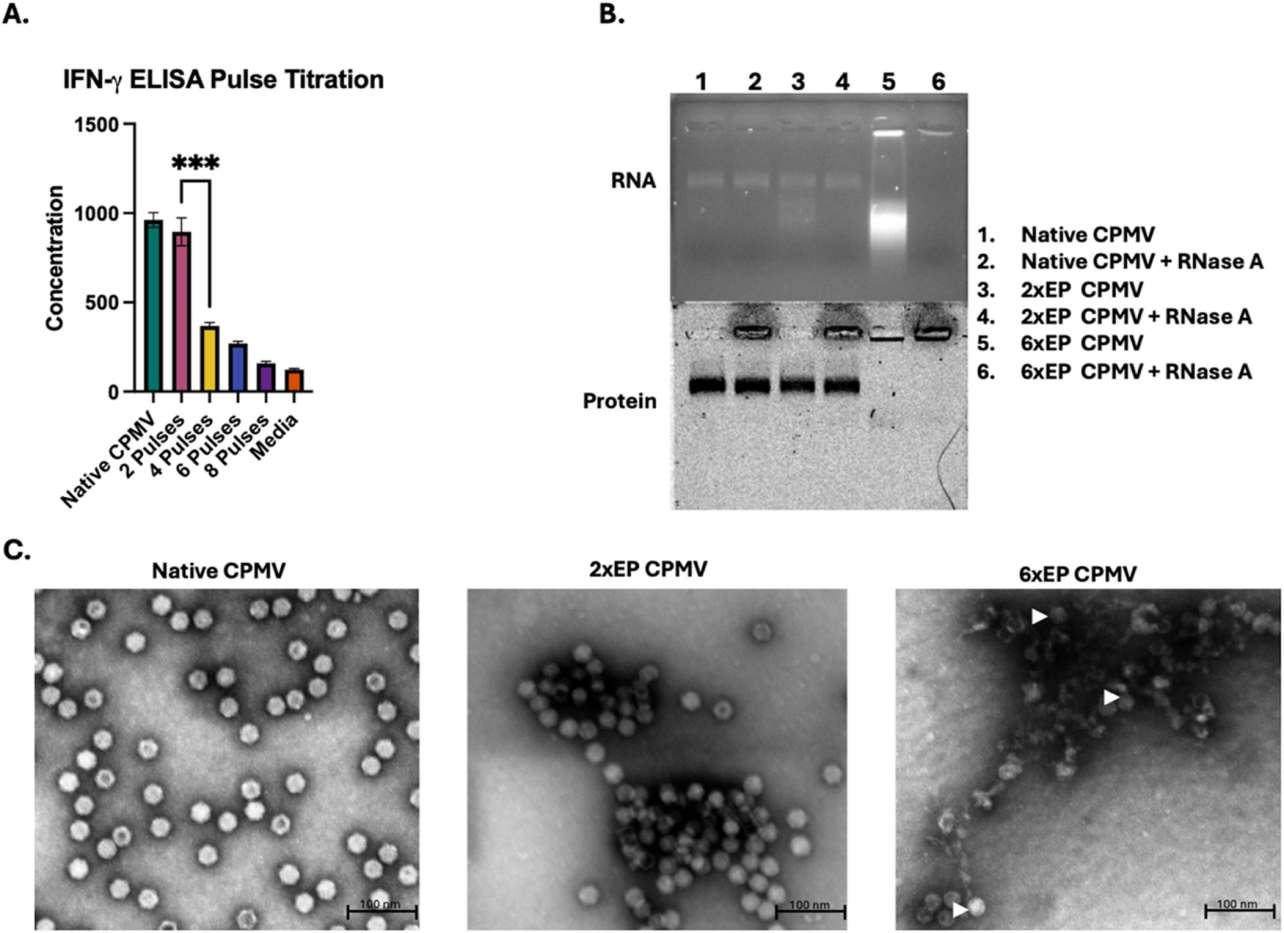
CPMV’s structure is disrupted by electroporation. (A) ELISA to detect IFN-γ in supernatants of splenocytes cultured *in vitro* with CPMV electroporated with varying pulses at 1500 V shows that CPMV maintains immunogenicity after 2 pulses but not after 4 or more pulses. The ELISA was repeated once with three samples per group, and representative data from the two experiments is shown here. (B) Agarose gel electrophoresis of CPMV stained with EtBr (top) to visualize RNA and Coomassie (bottom) to visualize protein. RNase A appears as a stain surrounding wells 2, 4, and 6 of the protein stain. Aggregated protein product of 6xEP CPMV appears as a dark band at the bottom of wells 5 and 6. The gel electrophoresis study was repeated once, and a representative image is shown here. (C) TEM images of Native CPMV, 2xEP CPMV, and 6xEP CPMV show aggregation and degradation of CPMV in a dose-dependent manner. Examples of the few remaining intact particles in the 6xEP CPMV are indicated with white arrowheads. Images from several fields were obtained in the TEM experiment, and representative images are presented here. Statistical significance is reported with *p* > 0.05 as ns, *p* < 0.05 as *, *p* < 0.01 as **, *p* < 0.001 as ***, and *p* < 0.0001 as ****.

Gel electrophoresis was carried out under nonreducing conditions with native CPMV, 2xEP CPMV, and 6xEP CPMV using a 1.2% (w/v) agarose gel. The viral RNA was visualized with ethidium bromide (Figure 3B, top), and the coat proteins were subsequently visualized by staining the same gel with Coomassie Brilliant Blue (Figure 3B, bottom). Native CPMV produces an RNA band that colocalizes with the protein band, indicating that both components of the virion are moving together as an intact particle. Samples were run in duplicate with one sample treated with RNase A (lanes 2, 4, and 6) prior to electrophoresis.

If the CPMV capsid is intact, the RNase treatment has no impact on the RNA within the capsid since the capsid precludes access of the RNase to the RNA within. However, when the capsid structure is disrupted by EP, the RNA is exposed and RNase can degrade it. This is demonstrated in Figure 3B. The RNA comigrates with the intact capsid (as evident by colocalization of the RNA and protein staining)—and does not change when RNase is added. In stark contrast, when the capsid structure is compromised, (partially) degraded RNA appears as a smear (not as a distinct band) below the capsid protein band. CPMV upon 2x pulses of electroporation (Figure 3B, lanes 3 and 4) is mildly compromised as evident by free RNA banding beneath the main RNA/protein band in lane 3 (not treated with RNase A). This indicates some breakdown or escape of RNA from the capsid structure. In lane 4 where this sample, 2xEP CPMV, is treated with RNase A, the RNA/protein band remains, indicating that some RNA is retained within the capsid, but the free RNA is degraded by the enzymatic action of the RNase. We conclude that while 2xEP treatments have some damaging effect on CPMV, the preparation is not entirely degraded, with some nucleoprotein assemblies remaining detectable by gel electrophoresis and TEM (see below).

As the number of pulses of electroporation is increased, particle damage becomes more severe. CPMV samples exposed to 6xEP (Figure 3B, lane 5) appear broken with no detectable migration of RNA with the capsid revealed by the protein gel—instead protein aggregation, which keeps the protein from uniformly migrating to form a band is apparent, with both RNA and protein detected in the well and the RNA released from the capsid. RNase treatment leads to degradation of the RNA as evident by loss of the RNA signal in the gels (Figure 3B, lanes 4 and 6). This increasing degree of damage is consistent with the electroporation titration ELISA.

The results were consistent when the virus particles were imaged by TEM (Figure 3C). Native CPMV appears as monodispersed 30 nm-sized hexagonal particles consistent with the icosahedral structure of the viral capsid. While the 2xEP CPMV samples appeared largely intact, there was evidence of aggregation, and the 6xEP CPMV samples were predominantly damaged particles with significant aggregation. Examples of the few remaining intact particles in the 6xEP + CPMV image are indicated with white arrowheads in Figure 3C.

Together, these data demonstrate that CPMV’s structure is damaged when electroporated with parameters used for *in vivo* plasmid electroporation. The degradation is dependent on the number of pulses of electroporation administered. We therefore conclude that ITIT combination therapy must apply the IL-12 plasmid electroporation prior to CPMV injection.

### CPMV and IL-12 Act Additively to Drive Robust Abscopal Immunity

To determine whether CPMV improves the ITIT local and abscopal response with IL-12 *in vivo* plasmid electroporation, we compared different sequences of IL-12 EP and CPMV injection using a two-tumor model. Mice were inoculated with either B16–F10 melanoma or MC38 colorectal cancer intradermal tumors on the right flank and then inoculated with tumors from the same cell line on the left flank 4 days later to establish a two-tumor mode. ITIT treatment was applied to the primary tumors (right flank) once per week for 2 weeks once they had reached sufficient volume (50 mm^3^). Mice were IT treated with PBS, CPMV (100 μg), IL-12 EP (*in vivo* electroporation of IL-12 plasmid, using six 1 ms pulses at 1500 V), CPMV + IL-12-EP (CPMV followed by *in vivo* electroporation of IL-12 plasmid), or IL-12-EP + CPMV (*in vivo* electroporation of IL-12 plasmid followed by IT injection of CPMV) (Figure 4).

**Figure 4:**
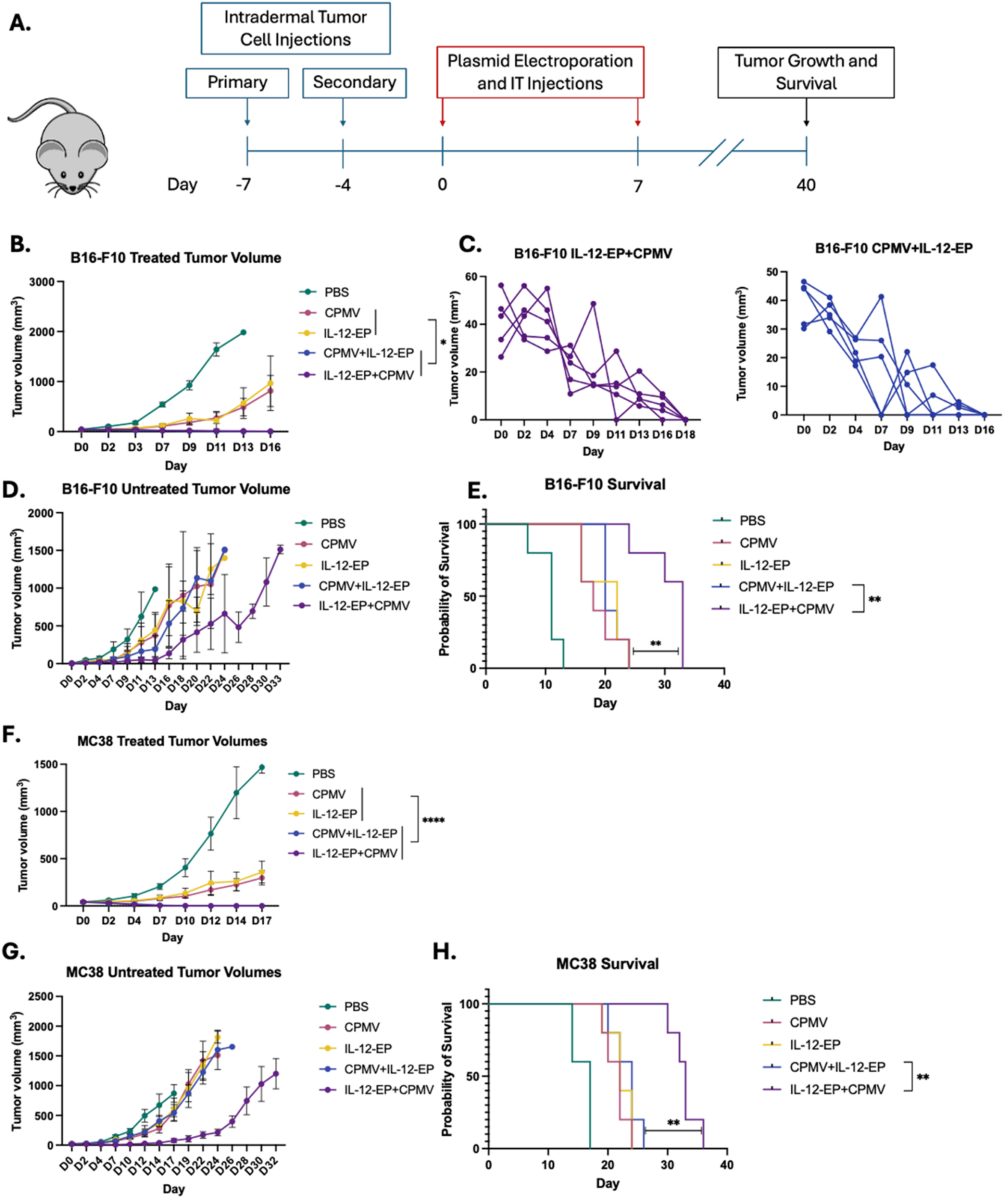
CPMV injection after IL-12 electroporation improves abscopal effect in the 2-tumor model. (A) Experimental timeline showing workflow of study. (B) All B16–F10 mice treated with CPMV followed by electroporation of IL-12 (CPMV + IL-12-EP) and all mice treated with electroporation of IL-12 followed by CPMV (IL-12-EP+CPMV) had clearance of the treated tumor. One mouse in the IL-12 monotherapy (IL-12-EP) and a mouse in the CPMV monotherapy group (CPMV) had clearance of the treated tumor. (C) Graphs showing volumes for treated tumors of mice in each dual therapy group. Each line represents the tumor growth for an individual mouse. (D) B16–F10 mice treated with CPMV alone (CPMV), IL-12 alone (IL-12-EP), and CPMV prior to IL-12 electroporation (CPMV + IL-12-EP) had slower growth of the untreated tumor than PBS. Mice treated with IL-12 electroporation prior to CPMV (IL-12-EP + CPMV) had the slowest growth of the untreated tumor. (E) B16–F10 mice treated with IL-12 electroporation prior to CPMV injection had longer survival owing to improved abscopal effect than any of the other treatment groups (*N* = 5). (F) All MC38 mice treated with CPMV followed by electroporation of IL-12 (CPMV + IL-12-EP) and all mice treated with electroporation of IL-12 followed by CPMV (IL-12-EP + CPMV) had clearance of the treated tumor. Mice treated with each monotherapy had significantly slower primary tumor growth than the control. (G) MC38 mice treated with CPMV alone (CPMV), IL-12 alone (IL-12-EP), and CPMV prior to IL-12 electroporation (CPMV + IL-12-EP) had slower growth of the untreated tumor than that of PBS. Mice treated with IL-12 electroporation prior to CPMV (IL-12-EP + CPMV) had the slowest growth of the untreated tumor. (H) MC38 mice treated with IL-12 electroporation prior to CPMV injection had longer survival owing to improved abscopal effect than any of the other treatment groups. (*N* = 5) The B16–F10 experiment was replicated with *N* = 3, and similar results were observed. The full-scale *N* = 5 experiment is shown here. The MC38 experiment was conducted in order to cross-validate the B16–F10 results in a separate tumor model and was therefore not replicated. Statistical significance is reported with *p* > 0.05 as ns, *p* < 0.05 as *, *p* < 0.01 as **, *p* < 0.001 as ***, and *p* < 0.0001 as ****.

Figure 4A shows a treatment timeline for mice in the abscopal studies. Consistent with results reported in Figure 1, tumors given ITIT with either IL-12 electroporation (IL-12-EP) or IT CPMV injection (CPMV) alone showed significantly slower growth compared to mice treated with PBS (Figure 4B,E). The combination of IL-12 electroporation and CPMV therapy (IL-12-EP + CPMV or CPMV + IL-12-EP), regardless of the sequence of EP relative to CPMV administration, significantly enhanced treatment efficacy with the primary tumor growth rate suppressed for both B16–F10 and MC38 (Figure 4B,C,F). In fact, all tumors treated with either dual therapy (IL-12-EP + CPMV and CPMV + IL-12-EP) cleared their primary tumor by day 18 in B16–F10 (Figure 4B,C) and by day 14 in MC 38 (Figure 4F). Interestingly, the tumor growth rate of the treated tumor in the CPMV+IL-12-EP treatment group matched that of IL-12-EP + CPMV for both cell lines. The clearance of treated tumors in the CPMV + IL-12-EP group indicates that even though electroporation treatment reduced the efficacy of CPMV (Figure 1), EP-exposed CPMV still enhances the suppression of the primary tumor growth compared to either monotherapy (IL-12-EP or CPMV alone).

When assaying for the abscopal effect (by assessing the untreated tumor) and overall survival, mice treated with CPMV followed by IL-12 electroporation (CPMV + IL-12-EP) did not experience enhanced growth retardation in the untreated tumor in either B16–F10 or MC38 (Figure 4D,G). End point criteria were met when one tumor reached a volume of 1500 mm^3^. For all mice besides those in the PBS groups, the untreated tumor reached end point criteria before the treated tumor. Therefore, the end point was effectively determined by the level of growth suppression of the untreated tumor. Since the dual therapy of CPMV injection prior to IL-12 electroporation (CPMV-IL-12-EP) did not enhance the abscopal effect compared to either monotherapy in either cell line (Figure 4D,G), overall survival matched that of either of the monotherapies (Figure 4E,H).

In stark contrast, enhanced potency of the IL-12-EP+CPMV groups, where CPMV is not exposed to electroporation, was observed in the distant, noninjected tumors: Both IT CPMV injection and IL-12 electroporation confer some abscopal effect in B16–F10 melanoma and MC38 colorectal cancer, but the growth retardation of the untreated tumor is significantly enhanced when CPMV follows IL-12 EP resulting in increased survival (Figure 4D,G). The IL-12 EP+CPMV combination therapy acts additively to suppress tumor growth, both locally (injected tumor) and systemically (nontreated tumors). This improved control over the untreated tumor translated to better survival for the groups treated with CPMV following IL-12 EP (CPMV + IL-12-EP) compared to all other groups in both cell lines.

## Discussion

The combination of IL-12 *in vivo* electroporation followed by IT injection of CPMV generates robust abscopal immunity that outperforms either monotherapy on its own. Previous studies have demonstrated either IL-12 plasmid electroporation^24,25^ or ITIT with CPMV^5–7,26^ are effective immunotherapies that generate an abscopal effect in models of B16–F10 melanoma and MC38 colorectal cancer. Our study shows that the abscopal effect can be improved if CPMV is injected after IL-12 electroporation is applied. While CPMV exposed to electroporation alone confers no efficacy (Figure 1), the addition of IL-12 conferred enhanced efficacy when comparing the CPMV + IL-12 EP group *vs* either treatment alone. This shows that the structural damage reduced but did not abolish CPMV efficacy, and some base level of immunomodulation activity remains that enhanced IL-12 therapy locally, but not systemically (Figure 4).

The most potent combination is the IL-12 EP + CPMV—here, CPMV is not exposed to electroporation, the capsid structure is not altered, and antitumor efficacy is maintained. The increased efficacy of the combination of IL-12 followed by CPMV ITIT can be explained because these therapies act along two separate axes, with CPMV activating innate immune cells and IL-12 priming a T-cell response, driving a more robust systemic immune response than is possible with either monotherapy on its own. While CPMV alone also generates increased IL-12 expression, this is variable;^27^ thus, the addition of IL-12 EP will further increase IL-12 expression ensuring robust and reproducible IL-12 signaling. Future studies documenting levels and timing of IL-12 comparing solo and combination therapies will improve our mechanistic understanding of the improved local and abscopal efficacy.

The *in vitro* exposure of splenocytes to CPMV (Figure 2) generated measurable IL-12, as opposed to prior reports of similar studies that did not identify IL-12 in similar experiments.^5^ What is clear from this report is that IL-12 plasmid electroporation clearly improved efficacy when combined with CPMV in comparison to IL-12 EP or CPMV by themselves.

Previous work has demonstrated the role of adaptive immunity in mediating the observed abscopal effects. The antitumor effects of IL-12 plasmid electroporation require both CD8+ T cells and IFN-γ signaling.^28^ Additionally, CPMV-based ITIT elicits abscopal suppression of untreated tumors through a CD8+ T-cell-dependent mechanism requiring cross-presentation by BatF3+ conventional dendritic cells (cDC 1s) and the efficacy of CPMV monotherapy is significantly reduced in the absence of endogenous IL-12 and IFN-γ.^5^ These prior findings, together with the replicated efficacy of our current dual therapy in both B16–F10 and MC38 models, provide strong support for a CD8+ T-cell-mediated systemic immune response induced by IL-12 EP followed by CPMV. Given the previous literature on the mechanism of IL-12 and CPMV efficacy separately, the data presented in this study strongly suggest that the improved abscopal effect is the result of amplified, antigen-specific T-cell immunity primed by the coordinated action of IL-12 and CPMV.

For any combination therapy, there is a need to evaluate timing, and here we determined that IL-12 EP must precede CPMV therapy to be effective. CPMV loses efficacy upon electroporation in a dose-dependent manner. CPMV’s immunomodulatory nature is linked to its repetitive pattern of the coat proteins and its packed ssRNA, which constitute pathogen-associated molecular patterns (PAMPs) recognizable by the mammalian immune system. Prior work using the bacteriophage Qβ showed that the virus-like particle’s immunogenicity is dependent on its particulate structure and that its immunogenicity is lost when it is disassembled into its individual coat proteins.^29^ Here we show that the electroporation compromises the structure of CPMV resulting in aggregation, particle breakage, and loss of RNA cargo, when the structure is compromised and CPMV immunogenicity is reduced (Figures 2 and 3).

While here we focused on a combination of CPMV with IL-12, the concept could be expanded to other chemo/cytokine therapies—for example, the proinflammatory cytokines, including TNF-a, IL-15, and IL-2, have shown promise in cancer therapy by slowing tumor growth mediated by an enhanced T-cell response in the TME.^30–32^ Again, because CPMV directly activates innate cells, leading to priming and expansion of antitumor T cells, the combination of CPMV with immunotherapies that act directly on T cells is expected to be additive and potentially synergistic.

While transfection of plasmid DNA encoding immunostimulatory cytokines has the potential to be used clinically, the technique may face limitations, as it may lead to inconsistent efficiency of plasmid uptake and expression of encoded proteins. To address these limitations, the use of lipid nanoparticles (LNPs) for the delivery of nucleic acids encoding proinflammatory cytokines is currently being investigated for immunotherapeutic use. LNP delivery of nucleic acid has been demonstrated to cause less tissue damage and to drive improved expression of the encoded protein product compared to *in vivo* electroporation,^33^ and IL-12 mRNA-loaded LNPs delivered intravenously into tumor-bearing mice have been demonstrated to significantly slow progression of cancer in preclinical models.^34,35^ Our study demonstrates that CPMV and IL-12 can be combined to bolster abscopal immunity, so the use of LNPs to deliver IL-12 into tumors could be used to further improve the efficacy of this approach. Further, IL-12 mRNA could be packaged into CPMV, combining the two therapies into a single treatment.

The combination of IL-12 and CPMV leads to a 100% local clearance of treated B16–F10 and MC38 tumors. The data show that CPMV bolsters the abscopal effect of IL-12 as a monotherapy when injected into the treated tumor after electroporation has already been applied. IL-12 electroporation has high clinical potential and is already being evaluated in Phase II clinical trials for the treatment of metastatic melanoma.^20^ The present study demonstrates a novel strategy to improve this already promising treatment by combining it with CPMV, leading to 100% elimination of the treated tumor and clearly improved control over the distant tumor. The powerful abscopal effect suggests that this treatment strategy has a direct translational value as an effective approach to metastatic disease. These studies highlight the necessity of initiating clinical trials to test this combination as an intratumoral immunotherapy.
